# The halogen-bonded adduct 1,4-bis(pyri­din-4-yl)buta-1,3-diyne–1,1,2,2,3,3,4,4,5,5,6,6,7,7,8,8-hexa­deca­fluoro-1,8-diiodo­octane (1/1)

**DOI:** 10.1107/S1600536813001888

**Published:** 2013-02-02

**Authors:** Gabriella Cavallo, Giovanni Marras, Pierangelo Metrangolo, Tullio Pilati, Giancarlo Terraneo

**Affiliations:** a NFMLab, Department of Chemistry, Materials and Chemical Engineering, "G. Natta", Politecnico di Milano, Via Mancinelli, 7, I-20131 Milano, Italy; b Chemessentia Srl, via Bovio 6, I-28100 Novara, Italy

## Abstract

In the crystal structure of the title compound, C_8_F_16_I_2_·C_14_H_8_N_2_, the mol­ecules form infinite chains parallel to [2-11] through two symmetry-independent C—I⋯N halogen bonds (XBs). As commonly found, the perfluoro­alkyl mol­ecules segregate from the hydro­carbon ones, forming a layered structure. Apart from the XBs, the only contact below the sum of van der Waals radii is a weak H⋯F contact. The topology of the network is a nice example of the paradigm of the expansion of ditopic starting modules; the XB leads to the construction of infinite supramolecular chains along [2-11] formed by alternating XB donors and acceptors.

## Related literature

For the use of bis-(4-pyrid­yl)buta-1,3-diine in crystal engeneering based on hydrogen bonding and transition metal binding, see: Nakamura *et al.* (2003 ▶); Curtis *et al.* (2005 ▶); Maekawa *et al.* (2000 ▶); Badruz Zaman *et al.* (2001 ▶); Allan *et al.* (1988 ▶). For N⋯I halogen bonds based on α,ω-diiodo­per­fluoro­carbons, see: Neukirch *et al.* (2005 ▶); Navarrini *et al.* (2000 ▶); Liantonio *et al.* (2003 ▶); Bertani *et al.* (2002 ▶); Metrangolo *et al.* (2004 ▶, 2008 ▶); Fox *et al.* (2004 ▶); Dey *et al.* (2009 ▶). For segregation of perfluoro­alkyl chains, see: Fox *et al.* (2008 ▶). For chirality and order/disorder of long perfluoro­alkyl chains, see: Monde *et al.* (2006 ▶). For the synthesis of bis-(4-pyrid­yl)buta-1,3-diine, see: Della Ciana & Haim (1984 ▶).
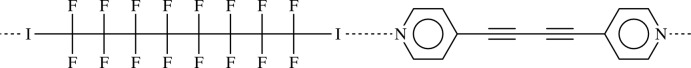


## Experimental

### 

#### Crystal data

C_8_F_16_I_2_·C_14_H_8_N_2_
*M* *_r_* = 858.10
Triclinic, 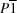

*a* = 5.4849 (11) Å
*b* = 14.302 (2) Å
*c* = 18.354 (3) Å
α = 111.40 (2)°
β = 90.35 (2)°
γ = 94.03 (2)°
*V* = 1336.4 (4) Å^3^
*Z* = 2
Mo *K*α radiation
μ = 2.48 mm^−1^
*T* = 295 K
0.36 × 0.12 × 0.10 mm

#### Data collection

Bruker SMART CCD area detector diffractometer
Absorption correction: multi-scan (*SADABS*; Bruker, 2010 ▶) *T*_min_ = 0.734, *T*_max_ = 1.000
15067 measured reflections
6100 independent reflections
4600 reflections with *I* > 2σ(*I*)
*R*_int_ = 0.026

#### Refinement

*R*[*F* ^2^ > 2σ(*F* ^2^)] = 0.039
*wR*(*F* ^2^) = 0.114
*S* = 1.02
6100 reflections
379 parameters
H-atom parameters constrained
Δρ_max_ = 0.90 e Å^−3^
Δρ_min_ = −0.57 e Å^−3^

### 

Data collection: *APEX2* (Bruker, 2010 ▶); cell refinement: *SAINT* (Bruker, 2010 ▶); data reduction: *SAINT*; program(s) used to solve structure: *SIR2002* (Burla *et al.*, 2003 ▶); program(s) used to refine structure: *SHELXL97* (Sheldrick, 2008 ▶); molecular graphics: *ORTEP-3 for Windows* (Farrugia, 2012 ▶) and *Mercury* (Macrae *et al.*, 2006 ▶); software used to prepare material for publication: *enCIFer* (Allen *et al.*, 2004 ▶).

## Supplementary Material

Crystal structure: contains datablock(s) global, I. DOI: 10.1107/S1600536813001888/fy2080sup1.cif

Structure factors: contains datablock(s) I. DOI: 10.1107/S1600536813001888/fy2080Isup2.hkl

Supplementary material file. DOI: 10.1107/S1600536813001888/fy2080Isup3.cml

Additional supplementary materials:  crystallographic information; 3D view; checkCIF report

## Figures and Tables

**Table 1:** Halogen and hydrogen-bonding contacts (Å, °)

C—*X*⋯*Y*	*X*⋯*Y*	C—*X*⋯*Y*
C1—I1⋯N1	2.863 (4)	177.93 (16)
C8—I2⋯N2^i^	2.887 (4)	175.39 (16)
C1—F1⋯H9^ii^	2.60	145.3

Symmetry codes: (i) = −2 + *x*, 1 + *y*, −1 + *z*; (ii) = −*x*, 1 − *y*, 1 − *z*.

**Fig. 1.**
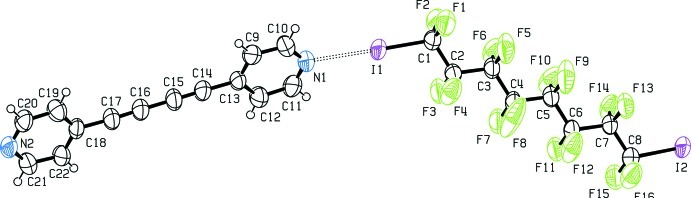
The asymmetric unit of the title compound. Displacement ellipsoids are drawn at 50% probability level.

**Fig. 2.**
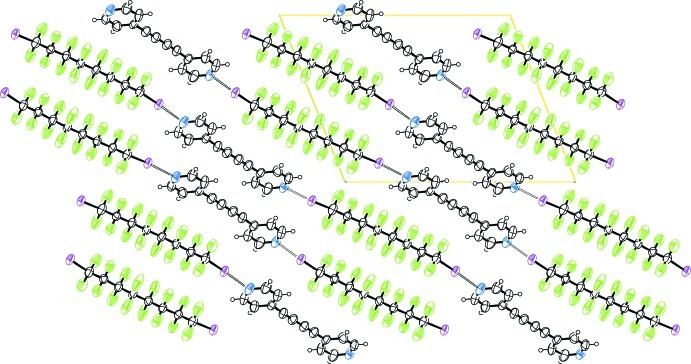
Crystal packing of the title compound viewed along the a axis, showing the alternating perfluorocarbon/hydrocarbon layers.

## supplementary crystallographic information

### Comment

Bis-(4-pyridyl)buta-1,3-diine (1) (Allan *et al.*, 1988) has been used as ditopic hydrogen bonding (HB) acceptor in crystal engineering (Nakamura *et al.*, 2003; Curtis *et al.*, 2005) and in transition metals complexes (Badruz Zaman *et al.*, 2001; Maekawa *et al.*, 2000), but it has never been used in halogen bonding (XB) adducts formation. Our group has shown that α,ω-diiodoperfluorocarbons are very good ditopic XB donors, both towards neutral (Fox *et al.*, 2004) and ionic (Metrangolo *et al.*, 2008) electron-donors. As expected, when solutions of (1) and 1,1,2,2,3,3,4,4,5,5,6,6,7,7,8,8-hexadecafluoro-1,8-diiodooctane (2), are mixed, the (1)···(2) adduct quickly precipitates (Fig. 1). It forms an infinite one-dimensional and non-covalent polymer through short XBs (Table 1). In our experience, this kind of nearly linear adduct has normally *Z*' = 1/2, that is, both molecules lie on crystallographic elements of symmetry, more frequently C_i_, but also C_2_ (see Table 2). Here instead, both molecules are in general position and *Z*' is 1. The cause of the molecular symmetry breaking is an F···H contact, the only interaction shorter than the sum of the van der Waals radii beside the I···N XBs. (Table 1). As happens in most structures containing perfluorocarbons and hydrocarbons moieties (see for example Fox *et al.*, 2008), the two components segregate and a layered structure is formed (Fig. 2).

### Experimental

(1) was synthetized according to Della Ciana & Haim (1984); (2) was from Aldrich. The adduct was obtained by slow evaporation from a 1:1 solution of the two components in chloroform.

### Refinement

The lowest energy conformation of long perfluoroalkanes is chiral in due to the sterically hindered F···F contacts between 1,3 positioned CF_2_ groups (Monde et al., 2006). Their crystals frequently show a superposition, in the same crystallographic site, of the two more common conformers: all-trans^+^ and all-trans^-^ (see, for example, Dey <i1,4-bis(pyridin-4-yl)buta-1,3-diyne–1,1,2,2,3,3,4,4,5,5,6,6,7,7,8,8-hexadecafluoro-1,8-diiodooctane (1/1)>et al., 2009), which in some cases have unequal occupancy factors. This is particularly visible for –CF_3_ terminated chains, that is, for α,ω-dihaloperfluorocarbons, which have the two endings strongly interacting with any electron-donor site. This type of disorder is difficult to observe, at least at room temperature, because it is masked by the large ADPs of perfluoroalkyl chains. This is due to the very weak interactions that their chains give with any environment. In the present study, splitting of some fluorine atoms was suggested by SHELXL but did not give good results. In spite of the use of a lot of restraints and constraints, at the price of a large increase of refined parameters, the final R_1_, wR_2_ and Δρ did not change significantly. The correlations between couples of parameters involving split atoms were very high, many of them being in the range 0.95–0.99. For these reasons we decided to use the ordered model of refinement. All H atoms were placed in geometrically calculated positions with C—H = 0.93 Å and U_iso_(H) = 1.2 U_eq_(C).

### Crystal data

**Table:**

C_8_F_16_I_2_·C_14_H_8_N_2_	*Z* = 2
*M**_r_* = 858.10	*F*(000) = 808
Triclinic, *P*1	*D*_x_ = 2.132 Mg m^−^^3^
*a* = 5.4849 (11) Å	Mo *K*α radiation, λ = 0.71073 Å
*b* = 14.302 (2) Å	Cell parameters from 981 reflections
*c* = 18.354 (3) Å	θ = 2.4–27.4°
α = 111.40 (2)°	µ = 2.48 mm^−^^1^
β = 90.35 (2)°	*T* = 295 K
γ = 94.03 (2)°	Elongated prism, colourless
*V* = 1336.4 (4) Å^3^	0.36 × 0.12 × 0.10 mm

### Data collection

**Table:**

Bruker SMART CCD area detector diffractometer	4600 reflections with *I* > 2σ(*I*)
ω and φ scans	*R*_int_ = 0.026
Absorption correction: multi-scan (*SADABS*; Bruker, 2010)	θ_max_ = 27.5°, θ_min_ = 2.3°
*T*_min_ = 0.734, *T*_max_ = 1.000	*h* = −7→7
15067 measured reflections	*k* = −18→18
6100 independent reflections	*l* = −23→23

### Refinement

**Table:**

Refinement on *F*^2^	0 restraints
Least-squares matrix: full	Hydrogen site location: inferred from neighbouring sites
*R*[*F*^2^ > 2σ(*F*^2^)] = 0.039	H-atom parameters constrained
*wR*(*F*^2^) = 0.114	*w* = 1/[σ^2^(*F*_o_^2^) + (0.0662*P*)^2^ + 0.5679*P*] where *P* = (*F*_o_^2^ + 2*F*_c_^2^)/3
*S* = 1.02	(Δ/σ)_max_ = 0.002
6100 reflections	Δρ_max_ = 0.90 e Å^−^^3^
379 parameters	Δρ_min_ = −0.57 e Å^−^^3^

### Special details

**Table:**

Geometry. All e.s.d.'s (except the e.s.d. in the dihedral angle between two l.s. planes) are estimated using the full covariance matrix. The cell e.s.d.'s are taken into account individually in the estimation of e.s.d.'s in distances, angles and torsion angles; correlations between e.s.d.'s in cell parameters are only used when they are defined by crystal symmetry. An approximate (isotropic) treatment of cell e.s.d.'s is used for estimating e.s.d.'s involving l.s. planes.

### Fractional atomic coordinates and isotropic or equivalent isotropic displacement parameters (Å^2^)

**Table:**

	*x*	*y*	*z*	*U*_iso_*/*U*_eq_	
I1	0.11335 (6)	0.46492 (2)	0.32421 (2)	0.06421 (11)	
C1	−0.0850 (8)	0.5404 (4)	0.2631 (3)	0.0616 (10)	
F1	−0.1957 (7)	0.6162 (3)	0.3136 (2)	0.1019 (12)	
F2	−0.2603 (6)	0.4751 (3)	0.2165 (2)	0.0938 (10)	
C2	0.0789 (7)	0.5809 (3)	0.2117 (2)	0.0525 (9)	
F3	0.2403 (6)	0.6512 (3)	0.25989 (19)	0.0936 (10)	
F4	0.1996 (6)	0.5068 (2)	0.16550 (18)	0.0852 (9)	
C3	−0.0565 (8)	0.6289 (3)	0.1613 (2)	0.0598 (10)	
F5	−0.1913 (8)	0.6966 (3)	0.2048 (2)	0.1254 (16)	
F6	−0.2101 (6)	0.5560 (3)	0.1115 (2)	0.1027 (12)	
C4	0.1092 (8)	0.6720 (3)	0.1114 (2)	0.0558 (9)	
F7	0.2517 (11)	0.7470 (4)	0.1598 (3)	0.170 (3)	
F8	0.2464 (7)	0.6031 (4)	0.0697 (3)	0.1272 (17)	
C5	−0.0268 (8)	0.7128 (3)	0.0561 (2)	0.0569 (10)	
F9	−0.1635 (10)	0.7821 (4)	0.0985 (2)	0.152 (2)	
F10	−0.1735 (7)	0.6388 (3)	0.0088 (2)	0.1142 (14)	
C6	0.1400 (8)	0.7555 (3)	0.0062 (2)	0.0546 (9)	
F11	0.2902 (7)	0.8298 (3)	0.0525 (2)	0.1111 (13)	
F12	0.2741 (6)	0.6821 (3)	−0.0373 (2)	0.0963 (11)	
C7	0.0006 (7)	0.7947 (3)	−0.0502 (2)	0.0519 (9)	
F13	−0.1322 (6)	0.8675 (2)	−0.00739 (17)	0.0850 (9)	
F14	−0.1497 (6)	0.7197 (2)	−0.09729 (17)	0.0826 (9)	
C8	0.1623 (9)	0.8356 (4)	−0.1016 (3)	0.0651 (11)	
F15	0.3155 (6)	0.9102 (3)	−0.0564 (2)	0.1065 (12)	
F16	0.2988 (7)	0.7622 (3)	−0.1455 (2)	0.1068 (13)	
I2	−0.04797 (6)	0.88682 (2)	−0.17707 (2)	0.06339 (11)	
C9	0.4885 (9)	0.3202 (4)	0.5119 (3)	0.0669 (12)	
H1B	0.4645	0.3284	0.5639	0.080*	
C10	0.3452 (9)	0.3656 (4)	0.4747 (3)	0.0758 (13)	
H10	0.2236	0.4041	0.5031	0.091*	
N1	0.3700 (8)	0.3578 (3)	0.4013 (3)	0.0730 (11)	
C11	0.5425 (10)	0.3021 (5)	0.3621 (3)	0.0775 (14)	
H11	0.5607	0.2952	0.3101	0.093*	
C12	0.6971 (9)	0.2536 (4)	0.3932 (3)	0.0689 (12)	
H12	0.8175	0.2160	0.3634	0.083*	
C13	0.6682 (7)	0.2623 (3)	0.4706 (2)	0.0553 (9)	
C14	0.8229 (8)	0.2136 (4)	0.5068 (3)	0.0616 (10)	
C15	0.9543 (9)	0.1761 (4)	0.5378 (3)	0.0651 (11)	
C16	1.1066 (9)	0.1343 (4)	0.5753 (3)	0.0646 (11)	
C17	1.2428 (9)	0.0982 (4)	0.6076 (3)	0.0654 (11)	
C18	1.3964 (8)	0.0545 (3)	0.6481 (2)	0.0565 (10)	
C19	1.3530 (9)	0.0648 (4)	0.7250 (3)	0.0678 (11)	
H19	1.2238	0.0998	0.7514	0.081*	
C20	1.5051 (9)	0.0220 (4)	0.7606 (3)	0.0674 (11)	
H20	1.4754	0.0291	0.8121	0.081*	
N2	1.6933 (7)	−0.0291 (3)	0.7268 (2)	0.0699 (10)	
C21	1.7289 (9)	−0.0398 (4)	0.6527 (3)	0.0743 (13)	
H21	1.8567	−0.0769	0.6274	0.089*	
C22	1.5882 (9)	0.0006 (4)	0.6113 (3)	0.0706 (13)	
H22	1.6214	−0.0082	0.5597	0.085*	

### Atomic displacement parameters (Å^2^)

**Table:**

	*U*^11^	*U*^22^	*U*^33^	*U*^12^	*U*^13^	*U*^23^
I1	0.06808 (19)	0.0752 (2)	0.06554 (19)	0.00504 (14)	−0.00501 (13)	0.04518 (15)
C1	0.065 (3)	0.071 (3)	0.062 (2)	0.017 (2)	0.009 (2)	0.037 (2)
F1	0.132 (3)	0.111 (2)	0.094 (2)	0.070 (2)	0.056 (2)	0.0628 (19)
F2	0.0727 (18)	0.119 (3)	0.116 (3)	−0.0210 (18)	−0.0270 (17)	0.079 (2)
C2	0.054 (2)	0.057 (2)	0.052 (2)	0.0110 (18)	0.0002 (17)	0.0243 (18)
F3	0.108 (2)	0.100 (2)	0.084 (2)	−0.0334 (19)	−0.0355 (18)	0.0554 (18)
F4	0.103 (2)	0.101 (2)	0.0805 (18)	0.0584 (18)	0.0315 (16)	0.0569 (16)
C3	0.068 (3)	0.067 (2)	0.056 (2)	0.021 (2)	0.006 (2)	0.034 (2)
F5	0.171 (4)	0.146 (3)	0.112 (3)	0.114 (3)	0.073 (3)	0.091 (2)
F6	0.091 (2)	0.135 (3)	0.110 (3)	−0.040 (2)	−0.0437 (19)	0.087 (2)
C4	0.061 (2)	0.058 (2)	0.060 (2)	0.0006 (19)	−0.0104 (18)	0.0354 (19)
F7	0.238 (5)	0.165 (4)	0.139 (3)	−0.132 (4)	−0.127 (4)	0.120 (3)
F8	0.122 (3)	0.180 (4)	0.158 (3)	0.098 (3)	0.080 (3)	0.138 (3)
C5	0.067 (2)	0.060 (2)	0.049 (2)	0.021 (2)	0.0048 (18)	0.0245 (18)
F9	0.222 (5)	0.187 (4)	0.122 (3)	0.158 (4)	0.109 (3)	0.118 (3)
F10	0.107 (3)	0.161 (3)	0.101 (2)	−0.058 (2)	−0.046 (2)	0.092 (3)
C6	0.061 (2)	0.057 (2)	0.053 (2)	0.0073 (18)	−0.0054 (18)	0.0279 (18)
F11	0.130 (3)	0.118 (3)	0.105 (2)	−0.055 (2)	−0.068 (2)	0.077 (2)
F12	0.101 (2)	0.128 (3)	0.102 (2)	0.069 (2)	0.0459 (18)	0.081 (2)
C7	0.057 (2)	0.054 (2)	0.050 (2)	0.0100 (17)	−0.0028 (17)	0.0253 (17)
F13	0.105 (2)	0.098 (2)	0.0755 (17)	0.0553 (18)	0.0257 (16)	0.0516 (16)
F14	0.095 (2)	0.0869 (19)	0.0769 (18)	−0.0257 (16)	−0.0342 (16)	0.0497 (15)
C8	0.062 (3)	0.079 (3)	0.070 (3)	0.009 (2)	−0.001 (2)	0.045 (2)
F15	0.093 (2)	0.131 (3)	0.123 (3)	−0.044 (2)	−0.047 (2)	0.089 (2)
F16	0.110 (2)	0.147 (3)	0.109 (2)	0.073 (2)	0.051 (2)	0.090 (2)
I2	0.07009 (19)	0.0747 (2)	0.06057 (18)	0.00691 (14)	−0.00418 (13)	0.04264 (15)
C9	0.061 (2)	0.090 (3)	0.062 (3)	0.006 (2)	0.000 (2)	0.042 (2)
C10	0.066 (3)	0.093 (3)	0.088 (3)	0.026 (3)	0.008 (2)	0.052 (3)
N1	0.064 (2)	0.091 (3)	0.086 (3)	0.011 (2)	−0.005 (2)	0.058 (2)
C11	0.078 (3)	0.110 (4)	0.066 (3)	0.006 (3)	−0.002 (2)	0.057 (3)
C12	0.068 (3)	0.087 (3)	0.063 (3)	0.017 (2)	0.004 (2)	0.038 (2)
C13	0.050 (2)	0.064 (2)	0.060 (2)	0.0001 (18)	−0.0102 (17)	0.034 (2)
C14	0.062 (2)	0.073 (3)	0.062 (2)	0.003 (2)	−0.0062 (19)	0.039 (2)
C15	0.067 (3)	0.069 (3)	0.070 (3)	0.010 (2)	−0.009 (2)	0.038 (2)
C16	0.067 (3)	0.071 (3)	0.067 (3)	0.008 (2)	−0.006 (2)	0.038 (2)
C17	0.066 (3)	0.071 (3)	0.069 (3)	0.010 (2)	−0.008 (2)	0.038 (2)
C18	0.061 (2)	0.057 (2)	0.062 (2)	0.0009 (18)	−0.0117 (19)	0.0348 (19)
C19	0.070 (3)	0.076 (3)	0.057 (2)	0.012 (2)	−0.007 (2)	0.023 (2)
C20	0.075 (3)	0.080 (3)	0.055 (2)	0.002 (2)	−0.010 (2)	0.036 (2)
N2	0.064 (2)	0.086 (3)	0.077 (3)	−0.001 (2)	−0.0163 (19)	0.051 (2)
C21	0.066 (3)	0.091 (3)	0.081 (3)	0.020 (3)	−0.002 (2)	0.047 (3)
C22	0.064 (3)	0.104 (4)	0.062 (3)	0.016 (3)	0.000 (2)	0.051 (3)

### Geometric parameters (Å, º)

**Table:**

I1—C1	2.154 (4)	C9—C10	1.376 (6)
C1—F1	1.330 (5)	C9—C13	1.377 (7)
C1—F2	1.345 (6)	C9—H1B	0.9300
C1—C2	1.540 (6)	C10—N1	1.320 (7)
C2—F4	1.316 (5)	C10—H10	0.9300
C2—F3	1.338 (5)	N1—C11	1.321 (7)
C2—C3	1.552 (5)	C11—C12	1.376 (7)
C3—F5	1.290 (5)	C11—H11	0.9300
C3—F6	1.344 (6)	C12—C13	1.390 (6)
C3—C4	1.545 (6)	C12—H12	0.9300
C4—F8	1.298 (6)	C13—C14	1.436 (6)
C4—F7	1.315 (5)	C14—C15	1.185 (6)
C4—C5	1.552 (6)	C15—C16	1.375 (6)
C5—F9	1.303 (5)	C16—C17	1.204 (6)
C5—F10	1.312 (6)	C17—C18	1.434 (6)
C5—C6	1.546 (6)	C18—C22	1.378 (7)
C6—F11	1.319 (5)	C18—C19	1.389 (6)
C6—F12	1.336 (5)	C19—C20	1.362 (6)
C6—C7	1.563 (5)	C19—H19	0.9300
C7—F13	1.322 (5)	C20—N2	1.330 (7)
C7—F14	1.330 (5)	C20—H20	0.9300
C7—C8	1.536 (6)	N2—C21	1.329 (6)
C8—F15	1.324 (6)	C21—C22	1.372 (6)
C8—F16	1.346 (6)	C21—H21	0.9300
C8—I2	2.151 (4)	C22—H22	0.9300
			
F1—C1—F2	107.3 (4)	F15—C8—F16	107.0 (4)
F1—C1—C2	109.0 (4)	F15—C8—C7	109.4 (4)
F2—C1—C2	108.1 (4)	F16—C8—C7	108.8 (4)
F1—C1—I1	110.5 (3)	F15—C8—I2	109.8 (3)
F2—C1—I1	108.9 (3)	F16—C8—I2	109.3 (3)
C2—C1—I1	112.9 (3)	C7—C8—I2	112.5 (3)
F4—C2—F3	108.4 (4)	C10—C9—C13	118.7 (4)
F4—C2—C1	108.5 (3)	C10—C9—H1B	120.6
F3—C2—C1	107.0 (3)	C13—C9—H1B	120.6
F4—C2—C3	109.1 (3)	N1—C10—C9	123.9 (5)
F3—C2—C3	108.2 (3)	N1—C10—H10	118.1
C1—C2—C3	115.4 (3)	C9—C10—H10	118.1
F5—C3—F6	106.3 (4)	C10—N1—C11	117.0 (4)
F5—C3—C4	110.6 (4)	N1—C11—C12	124.2 (5)
F6—C3—C4	107.1 (3)	N1—C11—H11	117.9
F5—C3—C2	110.1 (4)	C12—C11—H11	117.9
F6—C3—C2	107.0 (4)	C11—C12—C13	118.1 (5)
C4—C3—C2	115.2 (3)	C11—C12—H12	120.9
F8—C4—F7	108.2 (5)	C13—C12—H12	120.9
F8—C4—C3	109.2 (3)	C9—C13—C12	118.1 (4)
F7—C4—C3	107.6 (4)	C9—C13—C14	120.8 (4)
F8—C4—C5	108.6 (4)	C12—C13—C14	121.1 (4)
F7—C4—C5	107.7 (4)	C15—C14—C13	178.1 (5)
C3—C4—C5	115.4 (4)	C14—C15—C16	178.8 (6)
F9—C5—F10	107.2 (5)	C17—C16—C15	179.1 (6)
F9—C5—C6	109.6 (4)	C16—C17—C18	177.5 (5)
F10—C5—C6	108.5 (3)	C22—C18—C19	118.6 (4)
F9—C5—C4	108.2 (4)	C22—C18—C17	120.8 (4)
F10—C5—C4	107.9 (4)	C19—C18—C17	120.6 (4)
C6—C5—C4	115.2 (4)	C20—C19—C18	118.1 (5)
F11—C6—F12	108.1 (4)	C20—C19—H19	121.0
F11—C6—C5	109.6 (4)	C18—C19—H19	121.0
F12—C6—C5	107.9 (3)	N2—C20—C19	124.4 (4)
F11—C6—C7	108.4 (3)	N2—C20—H20	117.8
F12—C6—C7	108.0 (3)	C19—C20—H20	117.8
C5—C6—C7	114.7 (3)	C21—N2—C20	116.6 (4)
F13—C7—F14	108.4 (4)	N2—C21—C22	123.8 (5)
F13—C7—C8	108.1 (3)	N2—C21—H21	118.1
F14—C7—C8	107.8 (3)	C22—C21—H21	118.1
F13—C7—C6	108.2 (3)	C21—C22—C18	118.4 (4)
F14—C7—C6	108.5 (3)	C21—C22—H22	120.8
C8—C7—C6	115.6 (3)	C18—C22—H22	120.8
			
F1—C1—C2—F4	176.0 (4)	F10—C5—C6—F12	−62.3 (5)
F2—C1—C2—F4	−67.7 (4)	C4—C5—C6—F12	58.8 (5)
I1—C1—C2—F4	52.8 (4)	F9—C5—C6—C7	−58.7 (5)
F1—C1—C2—F3	59.2 (5)	F10—C5—C6—C7	58.1 (5)
F2—C1—C2—F3	175.5 (4)	C4—C5—C6—C7	179.1 (3)
I1—C1—C2—F3	−64.0 (4)	F11—C6—C7—F13	−63.1 (5)
F1—C1—C2—C3	−61.3 (5)	F12—C6—C7—F13	−179.9 (4)
F2—C1—C2—C3	55.0 (5)	C5—C6—C7—F13	59.8 (5)
I1—C1—C2—C3	175.5 (3)	F11—C6—C7—F14	179.6 (4)
F4—C2—C3—F5	175.0 (4)	F12—C6—C7—F14	62.7 (5)
F3—C2—C3—F5	−67.2 (5)	C5—C6—C7—F14	−57.6 (5)
C1—C2—C3—F5	52.6 (6)	F11—C6—C7—C8	58.3 (5)
F4—C2—C3—F6	59.9 (5)	F12—C6—C7—C8	−58.5 (5)
F3—C2—C3—F6	177.6 (4)	C5—C6—C7—C8	−178.8 (4)
C1—C2—C3—F6	−62.6 (5)	F13—C7—C8—F15	63.4 (5)
F4—C2—C3—C4	−59.1 (5)	F14—C7—C8—F15	−179.7 (4)
F3—C2—C3—C4	58.7 (5)	C6—C7—C8—F15	−58.0 (5)
C1—C2—C3—C4	178.5 (4)	F13—C7—C8—F16	180.0 (4)
F5—C3—C4—F8	178.5 (4)	F14—C7—C8—F16	−63.1 (4)
F6—C3—C4—F8	−66.0 (5)	C6—C7—C8—F16	58.5 (5)
C2—C3—C4—F8	52.9 (5)	F13—C7—C8—I2	−58.8 (4)
F5—C3—C4—F7	61.3 (6)	F14—C7—C8—I2	58.1 (4)
F6—C3—C4—F7	176.8 (4)	C6—C7—C8—I2	179.7 (3)
C2—C3—C4—F7	−64.3 (5)	C13—C9—C10—N1	−0.5 (8)
F5—C3—C4—C5	−58.9 (5)	C9—C10—N1—C11	0.6 (8)
F6—C3—C4—C5	56.6 (5)	C10—N1—C11—C12	−0.8 (8)
C2—C3—C4—C5	175.5 (4)	N1—C11—C12—C13	1.0 (9)
F8—C4—C5—F9	−179.8 (4)	C10—C9—C13—C12	0.6 (7)
F7—C4—C5—F9	−62.9 (6)	C10—C9—C13—C14	179.8 (5)
C3—C4—C5—F9	57.2 (6)	C11—C12—C13—C9	−0.8 (7)
F8—C4—C5—F10	64.5 (5)	C11—C12—C13—C14	179.9 (5)
F7—C4—C5—F10	−178.6 (4)	C22—C18—C19—C20	0.9 (7)
C3—C4—C5—F10	−58.5 (5)	C17—C18—C19—C20	−179.8 (5)
F8—C4—C5—C6	−56.9 (5)	C18—C19—C20—N2	0.0 (8)
F7—C4—C5—C6	60.1 (6)	C19—C20—N2—C21	−1.3 (8)
C3—C4—C5—C6	−179.8 (3)	C20—N2—C21—C22	1.7 (8)
F9—C5—C6—F11	63.5 (5)	N2—C21—C22—C18	−0.8 (9)
F10—C5—C6—F11	−179.7 (4)	C19—C18—C22—C21	−0.5 (7)
C4—C5—C6—F11	−58.7 (5)	C17—C18—C22—C21	−179.8 (5)
F9—C5—C6—F12	−179.0 (4)		

### Halogen and hydrogen-bonding contacts (Å, °).

**Table:**

C—*X*···*Y*	*X*···*Y*	C—*X*···*Y*
C1—I1···N1	2.863 (4)	177.93 (16)
C8—I2···N2^i^	2.887 (4)	175.39 (16)
C1—F1···H9^ii^	2.60	145.3

Symmetry codes: (i) = -2+x, 1+y, -1+z; (ii) = -x, 1-y, 1-z.

### Crystallographic symmetry site of single molecules in one-dimensional adducts between some molecules containg two basic N atoms and α,ω-diiodoperfluoroalkanes previously studied by our group.

**Table:**

Molecule A	Molecul B	A site	B site
C_14_H_8_N_2_^a^	I-(CF_2_)_8_-I	C_1_	C_1_
C_10_H_16_N_2_^b^	I-(CF_2_)_8_-I^c^	C_1_	C_1_
C_12_H_26_N_2_O_2_^d^	I-(CF_2_)_8_-I	C_i_	C_2_
C_10_H_16_N_2_^e^	Br-(CF_2_)_8_-Br	C_2_	C_i_
C_13_H_14_N_2_^f^	I-(CF_2_)_8_-I	C_i_	C_i_
CN-(CH_2_)_4_-CN^g^	I-(CF_2_)_2_-I	C_i_	C_i_
CN-(CH_2_)_4_-CN^g^	I-(CF_2_)_4_-I	C_i_	C_i_
CN-(CH_2_)_6_-CN^g^	I-(CF_2_)_4_-I	C_i_	C_i_
CN-(CH_2_)_4_-CN^g^	I-(CF_2_)_6_-I	C_i_	C_i_
CN-(CH_2_)_6_-CN^g^	I-(CF_2_)_6_-I	C_i_	C_i_
CN-(CH_2_)_4_-CN^g^	I-(CF_2_)_8_-I	C_i_	C_i_
CN-(CH_2_)_6_-CN^g^	I-(CF_2_)_8_-I	C_i_	C_i_
C_10_H_8_N_4_^h^	I-(CF_2_)_8_-I	C_1_	C_i_
C_10_H_8_N_4_^i^	I-(CF_2_)_4_-I	C_1_	C_i_

(a) This work. (b) N,N,N',N'-tetramethyl-p-phenylenediamine (Neukirch *et al.*, 2005). (c) Unusually, the conformation of I-(CF_2_)_8_-I is *ttgtt*. (d) 1,7,10,16-tetraoxa-4,13-diazacyclo-octadecane (Navarrini *et al.*, 2000). (e) 1,7,10,16-tetraoxa-4,13-diazacyclo-octadecane (Liantonio *et al.*, 2003). (f) 1,3-di-(4-pyridyl)propane (Bertani *et al.*, 2002). (g) Metrangolo *et al.* (2004). (h) 4,4'-azobispyridine (Fox *et al.*, 2004). (i) 1,7,10,16-tetraoxa-4,13-diazacyclo-octadecane (Dey *et al.*, 2009).
